# The effectiveness of metal on metal hip resurfacing: a systematic review of the available evidence published before 2002

**DOI:** 10.1186/1472-6963-4-39

**Published:** 2004-12-27

**Authors:** Laura Wyness, Luke Vale, Kirsty McCormack, Adrian Grant, Miriam Brazzelli

**Affiliations:** 1 Department of Public Health, University of Aberdeen, Aberdeen, UK; 2 Health Services Research Unit, University of Aberdeen, Aberdeen, UK; 3 Health Economics Research Unit, University of Aberdeen, Aberdeen, UK

## Abstract

**Background:**

Conventional total hip replacement (THR) may be felt to carry too high a risk of failure over a patient's lifetime, especially in young people. There is increasing interest in metal on metal hip resurfacing arthroplasty (MoM) as this offers a bone-conserving option for treating those patients who are not considered eligible for THR. We aim to evaluate the effectiveness of MoM for treatment of hip disease, and compare it with alternative treatments for hip disease offered within the UK.

**Methods:**

A systematic review was carried out to identify the relevant literature on MoM published before 2002. As watchful waiting and total hip replacement are alternative methods commonly used to alleviate the symptoms of degenerative joint disease of the hip, we compared MoM with these.

**Results:**

The data on the effectiveness of MoM are scarce, as it is a relatively new technique and at present only short-term results are available.

**Conclusion:**

It is not possible to make any firm conclusions about the effectiveness of MoM based on these early results. While the short-term results are promising, it is unclear if such results would be replicated in more rigorous studies, and what the long-term performance might be. Further research is needed which ideally should involve long-term randomised comparisons of MoM with alternative approaches to the clinical management of hip disease.

## Background

The treatment of younger people with disease of the hip joint presents a difficult clinical problem. Conventional total hip replacement (THR) may be felt to carry too high a risk of failure over a patient's lifetime. Overall, long-term results of THR in younger patients with a variety of underlying conditions indicate that 25–30% may require revision by 15 years [1], compared with less than five percent at ten years for older patients, and less than ten percent at ten or more years for all patients [2]. Specific subgroups of young active patients, such as those with osteoarthritis, may experience a revision rate of 50% [3]. In 1999/00 in the NHS in England 18% (8,389) of THRs were performed on people aged between 15 and 59, 46% (21,440) in people aged 60 to 74, and 36% (27,965) in people aged 75 and over [4]. Data on the number of revisions performed was not so readily available. A previous report suggested that out of approximately 2700 THRs per year, 2100 (78%) are primary THRs and 600 (22%) are revisions [5]. More recent data on revisions of THRs as a percentage of the total number of THR procedures suggest that in 1998/99 over ten percent of all THRs were carried out as revisions [6]. Due to concerns about the risks of revision, people who are expected to outlive a primary THR are often managed with non-surgical interventions, such medication to alleviate pain and to delay or prevent the need for surgery; collectively these interventions have been referred to as 'watchful waiting' (WW). People are typically referred for surgery only when their symptoms (e.g. pain, loss of physical function) become unmanageable by non-surgical means. Figures for the number of people who have their symptoms managed by pain control and other non-surgical interventions (such as the use of transcutaneous electrical nerve therapy and strengthening exercises) within England and Wales are difficult to determine. Evidence from a population survey suggest that 15.2 people per 1000 aged 35 to 85 years had hip disease severe enough for surgery. This equates to approximately 760,000 people within England and Wales [7].

Metal on metal hip resurfacing arthroplasty (MoM) offers a bone-conserving option for treating those patients who are not considered eligible for THR. MoM may also represent a more attractive alternative to other procedures such as osteotomy, bone fusion and arthroscopy, which have previously been used or been advocated as means of delaying or preventing the need for a THR. MoM involves the removal and replacement of the surface of the femoral head with a hollow metal hemisphere, which fits into a metal acetabular cup. This technique conserves femoral bone (although it is not conservative on the acetabular side), maintains normal femoral loading and stresses, and may not therefore compromise future total hip replacements. Data on the use of MoM within the NHS in England and Wales could not be obtained in this review. Never the less, because of increasing interest in MoM, we conducted a systematic review of the evidence of effectiveness aiming to compare it with THR and watchful waiting.

## Methods

### Search strategy

Initial searches failed to identify any randomised or comparative observational studies comparing MoM with any of the chosen alternatives. A structured search was conducted to identify evidence relating to the clinical effectiveness and cost-effectiveness of MoM for treatment of hip disease. The search strategy comprised of: (1) A free text search to identify any potentially relevant papers evaluating MoM (free text search terms were used because of the anticipated scarcity of published literature); and (2) A search for RCTs and systematic reviews of RCTs for THR using a modified version of the search strategy used for a recent review [8]. The search strategies used are presented in the appendix. Appendix [see Additional file 1] The following databases were searched to identify relevant published literature: Cochrane database of systematic reviews (CDSR), Database of abstracts of reviews of effectiveness (DARE), Cochrane Controlled Trials Register, MEDLINE and PREMEDLINE, EMBASE, HealthSTAR, CINAHL, NHS Economic Evaluation Database (EED), and Allied or Alternative Medicine (AMED). Relevant audit databases and the World-Wide Web were also searched. Unpublished data sources were sought by contacting experts in this field and industries with an interest in this area of orthopaedics. Studies from 1990 to 2001 were searched for.

### Inclusion and exclusion criteria

All identified abstracts were assessed for subject relevance independently by two reviewers. Full papers were then obtained and formally assessed for inclusion. It was agreed at the outset of the review that the search strategy would not be limited by language. It was agreed that non-English studies would be identified, but due to time and resource limitations would not be translated and assessed for their relevance to the review. No restrictions on the type of patient were imposed. Comprehensive systematic reviews of THR was carried out in Health Technology Assessment in 1998. These reviews were updated by the National Institute of Clinical Excellence (NICE) in 2000. Therefore, in this review a search for systematic reviews and RCTs published subsequent to the completion of the systematic reviews was carried out. Table 1 describes the inclusion and exclusion criteria applied for each of the treatments considered here.

### Data abstraction and quality assessment

Two reviewers independently abstracted data and quality assessed the included studies. Where a difference in opinion occurred, an arbiter was consulted. A data abstraction form was developed to record details of trial methods, participants, interventions, patient's characteristics and pre-specified outcomes (See Table 2). The quality assessment form was based on a checklist developed by Morris, 1988 [2] to assess the quality of studies appearing in orthopaedic research journals.

## Results

The initial search identified 352 potentially relevant MoM studies, 699 potentially relevant THR studies and 177 potentially relevant watchful studies. After reviewing titles and abstracts and applying the inclusion and exclusion criteria, data were abstracted from four published MoM studies [9-12], four published THR studies [2,8,13,14] and one watchful waiting study [15-17]. Four unpublished studies were also included [18-21]. These were obtained from companies that manufacture alternative MoM devices and also through personal communication with the Robert Jones and Agnes Hunt Orthopaedic and District Hospital.))No comparative studies were found.

### Quality of studies

The majority of studies rated poorly in terms of description of study sample, control of bias, and statistical and analytical considerations. Most studies rated favourably in terms of clarity of the study question and definition of outcome, although less favourably with respect to the description of the intervention. The duration and completeness of follow-up was of variable quality, in terms of the interval between surgery and follow-up being clearly stated and the consideration of patients lost to follow-up. Of the three systematic reviews included, two were of high quality [2,13], although there were some limitations on the comprehensiveness of the literature searches. The other systematic review was of lower quality with poor reporting of the methodology [8]. A summary of the quality assessment of the remaining ten included studies is presented in Table 3.

### Relative effectiveness of metal on metal hip resurfacing arthroplasty

#### Metal on metal hip resurfacing arthroplasty included studies

The MoM studies included in the review were four published studies, three unpublished reports from the manufactures of MoM prostheses, and one unpublished report. (Refer to table 4) The length of follow-up was less than five years for all the studies and ranging from 8.3 months [10] to 48 months [20]. The majority of the studies were small, (4424 [20] to four patients [11]). There was wide variation of patients' pre-operative diagnoses.

#### Metal on metal hip resurfacing arthroplasty study outcomes

Only one study reported details on the duration of the operation [11]. The mean operation time was reported as 247 minutes (range 180 to 370 minutes). McMinn et al, 1996 [10], reported that all patients were mobilised on the first post-operative day and at 12 days post-operation all patients had partial weight bearing of 25 kg on the surgically treated leg, with this weight being increased after 12 weeks. Patients in one study [12] spent a median of 21 days in hospital. All except one of the MoM studies reported the revision rates to THR. They ranged from 0% to 14.3%. Two groups of patients in the McMinn et al, 1996 [17] study were reported to have no revision to THR. Details on patients who were pain free were reported in one published study [17]. In this study 91% (60/66 patients) were pain free after a mean follow-up of 50.2 months (range 44 to 54 months). One of the manufacturers of MoM prostheses reported 71.1% (69/97 patients) to be pain free after a mean follow-up of 16.9 months [18].

The studies reported few complications. In one study [11] 10.5% (2/19 patients) were reported to have complications, one a femoral nerve palsy and one a haematoma. McMinn et al, 1996 [10] reported out of 235 patients, three patients had infections and one patient had sciatic nerve palsy. The only complication reported by Wagner et al, 1996 [12] (a study of 35 patients), was one patient with a femoral neck fracture, which was due to a traffic accident. The Oswestry Outcome Centre [20] reported the majority of revision surgery was due to fractures (56%), followed by loosening (19%), infection (11%), avascular necrosis (11%) and dislocation (3%). One manufacturer reported 6.4% (7/110 patients) to have complications [18]. Another manufacturer reported 3% (3/100 patients) to have complications [19]. The most common type of complication in these two studies was loosening.

### Alternative treatments to MoM

Only one watchful waiting study was included in this review. (Refer to table 5) The results of the study were reported in two papers, one with results up to three years [16] and the other up to eight years [17]. All the patients included in the study suffered from osteoarthritis of the hip. The study reported that the THR surgery performed increased from 9 patients (32%) at 3 years, to 14 patients (48%) at eight years. The number of patients using walking aids also increased from 8 patients (29%) at three years, to 12 patients (41%) at eight years. Patients' level of pain showed a slight increase from three to eight years.

Three systematic reviews provided the majority of information on THR for this review [2,8,13]. One of these reviews [2] included 11 RCTs (mean sample 168 patients), 18 comparative observational studies including two very large studies based on Scandinavian registry data [22], and 159 observational studies. The second systematic review [13] included 17 RCTs, 61 comparative studies and 145 observational studies. The third review [8] included the two systematic reviews mentioned above in addition to four RCTs, ten prospective comparative observational studies and Swedish Registry data [22]. One additional recent RCT [14] not included in the earlier systematic reviews was found from the search in this review. (Refer to table 6) The review by Fitzpatrick et al 1998 [2], reported an adjusted revision rate per 100 person years at risk of 0.37(+/- 0.02). Faulkner et al, 1998 [13] reported that cemented designs show good survival at ten to 15 years. The review by NICE, 2001 [8] reported that a number of prostheses achieved a revision rate of 10% or less after ten or more years follow-up. The study by Sharp et al, 2000 [14] reported a revision rate of 27.5% at a mean follow-up of 5.2 years. It was also reported in this study that two out of 91 patients (2.2%) had a dislocation within one year post-operation. No evidence on the extent or nature of complications was reported in any of the systematic reviews.

## Discussion

Despite extensive searching for relevant studies, the evidence base for making comparisons between MoM and any of the comparators is limited. Initial searches had already shown a lack of comparative studies and therefore the focus of the literature search was on identifying less methodologically robust studies such as data from case series. Although such searches are problematic due to lack of specific indexing terms, an extensive search strategy was devised to identify as many eligible studies as possible.

The early data pertaining to MoM suggests that MoM has the potential to be an effective technique for the management of hip disease. However, due to the lack of any controlled studies, it is difficult to know how much more or less effective it is compared to any comparators. The data available with which to make comparisons is uncontrolled and the studies identified have, in many cases, considered patient populations that are dissimilar in many ways. Identified studies also did not always use comparable outcomes and had different lengths of follow-up.

The lack of long-term data on MoM makes it difficult to compare with the other comparators. In particular the failure rates for some types of THR prosthesis increase significantly after ten years [2], and it is possible the same could occur with MoM. It is also unclear whether the success rates reported for THR could be replicated in younger or more active populations. Comparisons between MoM and THR studies are difficult as the MoM studies included younger patients and had shorter follow-up than the THR studies. The evidence from the systematic reviews of different methods of THR reported that several prostheses had revision rates of ten percent or less at ten years or more [2,13]. Revision rates reported in the MoM studies ranged from 0% to 14% for up to 5 years follow-up. The only other outcome that could be compared is the percentage of patients who were pain-free at follow-up. This was reported to be 90.9% at 50.2 months follow-up in one group of patients in one MoM study [10]. The systematic review conducted by Fitzpatrick and colleagues in 1998 report a mean of 84.1% (range 46–100%) of patients pain-free at a follow-up of 11 years [2].

In the MoM and WW studies, most of the patients had a preoperative diagnosis of osteoarthritis and were all of a similar younger age. The watchful waiting study reported 32% of patients requiring surgery at 3 years and 48% by eight years follow-up [15-17]. In the MoM studies revision rates ranged from 0% to 14.3%, after a follow-up of less than five years. During the 8-year follow-up period, people managed with WW had a slight increase in their pain levels, whereas the MoM patients hip scores all improved. 91% (60/66) of MoM patients were pain free after a mean follow-up of 50.2 months in one study [10], and 71% (69/97) after a mean follow-up of 16.9 months in the only other study that reported this outcome [18]. The very limited evidence available suggests that MoM is more effective in terms of better quality of life (measured by pain scores for WW and hip scores for MoM) than WW over a follow-up of approximately three years.

As the relative effectiveness of MoM is unclear the cost-effectiveness of MoM is also uncertain. It is likely the MoM procedure would cost approximately £5,500 whereas a THR would cost about £4,200 and the annual cost of WW (including the cost of NSAID (Non steroidal anti-inflammatory drugs) therapy, physiotherapy and treatment of side effects of medications) would be about £640 [23]. Whether MoM proves to be cost-effective against these alternatives depends upon the rates of revision to THR of MoM and WW, and the rates of revision of THR. The operation rates reported from the one WW study [15-17] and the revision rates of MoM suggest that MoM may provide better outcome at lower cost over a ten-year period. Such information remains at best tentative due to the small number of people to whom the watchful waiting data relate, the short follow-up of the MoM studies, and the uncontrolled nature of the comparison.

## Conclusions

The use of MoM in the UK is still relatively rare. However, there has been increasing interest from younger people with hip disease who are not currently considered eligible for THR and amongst surgeons who strive for better ways to treat the patients whom they see. However, only very limited evidence are currently available on MoM and although the procedure does appear promising the lack of robust comparisons with the other treatment options and of long term data make it virtually impossible to draw robust conclusions about its relative effectiveness. Given the early promise shown by MoM there is a real need for more rigorous research. Such research would be challenging, not least because of ethical considerations, but should attempt some form of prospective, preferably randomised, comparison of MoM with a policy of delayed selective surgery. These studies should preferably be large-scale, long-term, and use standard outcome measures, both pre- and post-operatively.

## Competing interests

The author(s) declare that they have no competing interests.

## Authors' contributions

LW carried out the critical appraisal of the included studies and assisted in the writing up. LV coordinated the project and assisted in the writing up. KM developed the methodology for the literature search and assisted in the writing up. AG participated in the design and coordination of the study. MB assisted in the critical appraisal of the included studies. All authors read and approved the final manuscript.

## Pre-publication history

The pre-publication history for this paper can be accessed here:



## Supplementary Material

Additional File 1 Search strategies. The search strategies used to search electronic databases to identify studies relevant to this review. Click here for file

## Figures and Tables

**Table 1:** Inclusion and exclusion criteria

Treatment	Inclusion/Exclusion Criteria
Metal on metal hip resurfacing	A minimum of two years follow-up was applied; Studies not reporting the specified outcomes (ref. table 2) such as laboratory only studies were excluded.
Watchful waiting	Observational data of people receiving WW with a follow-up of greater than five years were included
Total hip replacement	RCTs or systematic reviews of RCTs with a minimum of five years follow-up of different methods of THR and systematic reviews of such trials

**Table 2:** Outcomes sought from all included studies

Timescale	Outcomes
Short term	Duration of operation Serious complications (e.g. nerve palsy, haematoma dislocation, infection, re-operation within 6 months Time in hospital Time to return to "normal activities" prior to operation
Long term	Revision rate Time to revision surgery Functional result Percentage of patients pain free Quality of life (any recognised generic or condition specific measure e.g. SF-36) Mortality

**Table 3:** The number of studies identified for different treatments

	**Number of studies**			
**Assessment Item**	**Yes**	**No**	**Unable to judge**	**Not applicable**

**Clarity of study question and definition of outcome**				
Is the purpose of the study clearly stated?	8	1	1	-
Is the definition of prosthesis failure clear?	7	2	-	1
Is there a clear definition of primary outcome(s)?	7	3	-	-
Are standardised outcome measures used?	9	-	1	-
Are the outcome measures used appropriate for the purpose of the study?	9	1	-	-
**Description of prosthesis and method of fixation**				
Is the prosthesis design adequately described?	7	2	-	1
Is the method of fixation adequately described?	7	2	-	1
**Description of study sample**				
Is the method of selection of the sample adequately described?	2	7	1	-
Are the study exclusion and inclusion criteria stated?	2	7	1	-
Is the baseline sample clearly described in terms of basic characteristics (age, sex etc)?	6	3	1	-
Is the study sample sufficiently homogenous in terms of disease/diagnosis?	5	1	4	-
Is the study sample sufficiently homogenous in terms of co-morbidity?	2	-	8	-
**Control of bias in study design**				
Is the method of randomisation adequate?	-	-	1	9
Is the method of masking the patient to the intervention allocated stated?	-	2	1	7
Were outcome assessors blind to intervention allocation?	-	1	3	6
Are baseline values for groups compared?	-	1	1	8
Has the study adequately controlled for confounding factors?	1	7	2	-
**Duration and completeness of follow-up**				
Are intervals between surgery and follow-up assessment clearly stated?	8	2	-	-
Are reasons for loss of patients at follow-up stated?	2	2	2	4
Are those lost to follow-up compared to the rest of the sample?	1	2	3	4
Is there an appropriate length of follow-up?	8	2	-	-
Is the length of follow-up at least 5 years?	3	7	-	-
**Statistical and analytical considerations**				
Has the study sample size been justified?	-	9	1	-
Are the data clearly presented?	8	2	-	-
Was the data analyst masked to interventions?	-	5	3	2
Has type of statistical test and actual probability value been stated?	2	1	1	6
Are statistical tests appropriate to study?	2	-	2	6
Is the sample on which failures are assessed adequate?	-	2	7	1
Are conclusions justified by evidence?	5	1	3	1

**Table 4:** Metal-on-metal studies included in review.

Study (Study design) Funding source	Prostheses	Mean duration of follow up (range)	Mean patient age (range)	Revision Rate (unless otherwise stated)	Hip Score Pre-operation/Post-operation
Amstutz (2000) [9] Specialist orthopedic hospital, USA. (Observational) Not reported	Cemented, modified McMinn acetabular (7 Hips) Conserve Plus (29 Hips)	22 months (NR)	40 years (NR)	NR	NR
McMinn (1996) [10] 3 Birmingham Hospitals, UK. (Observational) Not reported	Uncemented, Uncoated (70 Hips)	50.2 months (44–54)^a^	48.7 years (NR)	8/66 patients = 12.1% 60/66 = 90.9% patients pain free	pain 3.1^b^/5.3 mobility 3.1^b^/5.3 walking 3.1^b^/5.3
	Uncemented, Hydroxyapetite coating (6 Hips)	40.2 months (38–42)^a^		0/6 patients = 0%	pain 3.0^b^/5.5 mobility 3.1^b^/6.0 walking 2.7^b^/5.7
	Cemented acetabular (43 Hips)	33.2 months (23–38)^a^		4/39 patients = 10.3%	pain 2.9^b^/5.4 mobility 3.0^b^/5.4 walking 3.2^b^/5.4
	Cemented acetabular, Hydroxyapetite coating (116 Hips)	8.3 months (1–19)^a^		0/109 patients = 0%	pain 3.0^b^/5.7 mobility 3.2^b^/5.7 walking 3.2^b^/5.7
Schmalzried (1996) [11] Specialist orthopedic hospital, USA. (Observational) Not reported	Cementless Wagner (4 Hips) Cemented McMinn (17 Hips)	16 months (10–25)	42 years (22–64)^a^	1/19 patients = 5.3%	pain 4^c^/9 walking 6^c^/9 function 6^c^/9 activity 4^cd^/7
Wagner (1996) [12] Specialist orthopedic hospital, Germany. (Observational) Not reported	2 Ti pins on cupshell (12 Hips) Press fit version (23 Hips)	20 months (6–54)	36 years (15–64)^a^	5/35 patients = 14.3%	32 (5–51)^e^ d/94 (72–100)^ed^
Corin Group Ltd, 2001 [18] 4 UK Hospitals, 1 UK clinic, 3 surgeons (Industry submission) Corin Group Ltd	Cormet 2000	21.36 months^f^	50.8 years (26–69)^f^	Revision Rate CIC 69/97 = 71.1%^f^ Patients pain free	NR
Midland Medical Technologies Ltd, 2001 [19] Hospitals in Birmingham Southampton Liverpool and Belgium (Industry submission) Midland Medical Technologies Ltd	Birmingham Hip Resurfacing (1761 Patients)	NR	49.2 years (15–86)	8/1382 hips = 0.6%	NR
Wright Cremascoli Ortho Ltd, 2001 [21] (Industry submission) Wright Cremascoli	Conserve Plus (100 Hips)	NR (24–51.6)	NR	3/100 hips = 3%	NR
Oswestry Outcome Centre Database [20] Oswestry Outcome Centre (Unpublished observational data) Oswestry Outcome Centre	McMinn (1378 Hips) All consultants (4424 Hips)	0–4 years 0–4 years	53.1 (NR) 49.2 (NR)	7/1378 = 0.5% 34/4424 = 0.77%	66.2^e^/98.1^e^ 61.3^e^/95.9^e^

a Median (range)

b Charnley hip score

c UCLA hip score

d Results of groups reported together

e Harris hip score

f Data from 97 patients (110 hips);

CIC – Data marked as "Commercial in confidence" in the industry report

NR – Not Reported

**Table 5:** Watchful waiting studies

Study (Study Design) Funding source	Patient Characteristics	Patients pain level at baseline	Patients pain level at follow-up	Use of walking aids	Notes
Dieppe (1997) [16] Single orthopedic unit (UK) Follow up: Mean (range) 37.6 months (31–41) (Observational study Arthritis and Rheumatism Council	N = 84 patients Mean age (SD): 50 (12.1)	None = 7% Mild = 48% Moderate = 31% Severe = 10%	None = 4% Mild = 50% Moderate = 32% Severe = 14%	9 patients (32 %) at baseline 8 patients (29%) at 3 year follow up	All patients had symptomatic limb joint osteoarthritis. Surgery performed in 9 patients (32%)
Dieppe (2000) [17] Single orthopedic unit (UK) Follow up: Mean (range) NR (36–96) (Observational study) Arthritis and Rheumatism Council	N = 29 patients Mean age (SD): 50 (12.1)	None = 7% Mild = 48% Moderate = 31% Severe = 10%	None = 3% Mild = 34% Moderate = 48% Severe = 14%	12 patients (41%) at 8 year follow up	All patients had symptomatic limb joint osteoarthritis. Surgery performed in 14 patients (48%)

Note: The studies above involve the same population, but have different length of follow up.

NR: Not Reported.

**Table 6:** Total hip replacement RCTs of 5 years or more duration not included in the systematic reviews [2,8,13].

Study (Study Design)	Prostheses	Patient Characteristics	Mean duration of follow up (range)	Revision Rate^bc^	Hip score post-op^d^
Sharp (2000) [14] 2 Hospitals (UK)^a ^(Observational study) No funding received	C-Fit uncemented with hydroxyapatite porous coating of components	N = 91 in total for both groups Mean age: <66 years	5.2 years (1 month–8 yrs)	25/91 = 27.5%	score/patients 12–20/35 21–30/13 31–40/12 41–50/1 50–60/2

a: 1 center randomised, 1 center not randomised

b: Revision rate at latest follow-up point & crude survival rate based on all patients

c: Results reported by patient number on entry to trial i.e. intention to treat

d: Oxford Hip Score

NR: Not Reported
