# Supplementary material for: The effectiveness of metal on metal hip resurfacing: a systematic review of the available evidence published before 2002
Source: BMC Health Serv Res. 2004 Dec 27;4:39. doi: 10.1186/1472-6963-4-39 (PMC544574; doi:10.1186/1472-6963-4-39)
Supplement: Additional File 1 — Search strategies. The search strategies used to search electronic databases to identify studies relevant to this review. [file 1472-6963-4-39-S1.doc]

**Appendix: Search strategies**

Metal on metal search strategy

1. (resurfac$ adj25 hip$).tw.
2. (surface adj5 replacement$ adj25 hip$).tw.
3. (resurfac$ adj25 prosthesis adj25 hip$).tw.
4. (surface adj5 replacement$ adj25 prosthesis adj25 hip$).tw.
5. (resurfac$ adj25 femoral head$).tw.
6. (surfac$ adj5 replacement$ adj25 femoral head$).tw.

7. (metal adj1 metal adj25 hip$ adj25 surface).tw.
7. (metal adj1 metal adj25 hip$ adj25 resurfac$).tw.
8. (cup adj25 resurfac$).tw.
9. (cup adj25 surface adj5 replacement$).tw.
10. or/1-10
11. animal/
12. human/
13. 11 and 12

14. 11 not 13
15. 10 not 14

Total Hip Replacement search strategy

1. controlled clinical trial.pt.

2. randomised controlled trial.pt.

3. randomised controlled trials/

4. random allocation/

5. double blind method/

6. single blind method/

7. or/1-6

8. (animal not human).sh

9. clinical trial.pt.

10. exp clinical trials/

11. (clin$ adj25 trial$).ti,ab.

12. ((singl$ or doubl$ or trebl$ or tripl$) adj25 (blind$ or mask$)).ti,ab.

13. placebos.sh.

14. placebo$.ti,ab.

15. random$.ti,ab.

16. research design.sh.

17. or/9-16

18. 7 or 17

19. 18 not 8

20. hip prosthesis/

21. prosthesis failure/

22. cementation/

23. reoperation/

24. exp prosthesis design/

25. prosthesis-related infections/

26. prosthesis fitting/

27. or/20-26

28. 19 and 27

29. limit 28 to yr = 1999-2001

Comparators search strategy

1. arthrodesis/ or arthroscopy/ or osteotomy.tw.
2. (hip or hips).tw.
3. (fem$ or head$).tw.
4. 2 or 3
5. 1 and 4
6. (bone$ adj1 (fusion or fusing or fused)).tw.
7. (joint$ adj1 (fusion or fusing or fused)).tw.
8. 6 or 7
9. 4 and 8
10. (arthroscop$ or osteotomy$ or arthrodes$).tw.
11.4 and 10
12. 5 or 9 or 11
13. limit 12 to yr = 1998 - 2001
